# Outcomes of Top-Down Holmium Laser Enucleation of Prostate for Recurrent/Residual Benign Prostatic Hyperplasia: One-Year Follow-Up

**DOI:** 10.1155/2022/5185114

**Published:** 2022-09-20

**Authors:** Ahmed S. Zakaria, Amr Hodhod, Loay Abbas, Moustafa Fathy, Ruba Abdul Hadi, Waleed Shabana, Anastasia Alexandra MacDonald, Ahmed Gamaleldin, Mohamed Abdallah, Mohamed Elgharbawy, Abdulrahman Ahmad, Adam Roos, Ahmed Kotb, Walid Shahrour, Hazem Elmansy

**Affiliations:** ^1^Urology Department, Northern Ontario School of Medicine, Thunder Bay, Ontario, Canada; ^2^Urology Department, Menoufia University, Shebin Elkom, Egypt

## Abstract

**Materials and Methods:**

We carried out a retrospective analysis of patients who underwent top-down HoLEP for the management of recurrent BPH at our institution. Patients who had previously undergone TURP were assigned to group I, while those with no history of prostate surgery were allocated to group II. Preoperative clinical characteristics, enucleation time, resected tissue weight, morcellation time, energy used, and intraoperative and postoperative complications were recorded and statistically analyzed. Patients were followed up postoperatively at 1, 3, 6, and 12 months. The evaluation included the International Prostate Symptom Score (IPSS), quality of life assessment (QoL), maximum urinary flow rate (*Q*_max_), postvoid residual urine test (PVR), and continence status.

**Results:**

Two hundred and sixty-nine patients were included in this study. Group I consisted of 68 patients with recurrent BPH, while group II included 201 patients. There were no statistically significant differences in preoperative characteristics between both groups. The median enucleation time for group I (67.5 min (25–200)) was not significantly longer than that for group II (60 min (19–165) (*p*=0.25)). Operative outcomes, including morcellation time, resected weight, catheter duration, and hospital stay, were comparable between both groups. At 1, 3, 6, and 12 months, all urinary functional outcomes showed significant improvement, and there were no significant differences between the two groups. At 3 months' follow-up, two patients in group I and three patients in group II experienced stress urinary incontinence (SUI). At the last follow-up visit, one patient from group I presented with persistent SUI.

**Conclusions:**

For managing recurrent and nonrecurrent cases of BPH, top-down HoLEP is safe with comparable urinary functional outcomes. Patients with a history of previous prostate surgery can be counselled that their prior transurethral procedure does not reduce the benefits of HoLEP.

## 1. Introduction

Transurethral resection of the prostate (TURP) is considered the gold standard surgical treatment for symptomatic benign prostatic hyperplasia (BPH). However, the seven-year retreatment rate of TURP ranges from 3 to 17.6% [1–3], with inadequate tissue removal and the progressive nature of the BPH being cited as the main reasons for reoperation [1].

Holmium laser enucleation of the prostate (HoLEP) is a safe and effective treatment option for patients experiencing lower urinary tract symptoms (LUTS). The HoLEP procedure has comparable outcomes to open prostatectomy (OP) and TURP, with a low morbidity rate and shorter hospital stay [2,4–7]. Improvements in outcome parameters following HoLEP are durable, and the late complication and reoperation rates are very low, up to 18 years [8]. However, many studies excluded patients with a history of prior prostate surgery, including TURP, as the surgical treatment of recurrent prostatic obstruction following previous transurethral surgery is more challenging due to the loss of anatomical landmarks [9].

Studies of HoLEP in patients with recurrent BPH demonstrate that the surgical plane between the adenoma and the surgical capsule remains accessible, thus producing durable long-term functional outcomes with minimal complications [9–12]. Many HoLEP techniques have been described in the literature, including top-down HoLEP [13–15]. The top-down technique is an anteroposterior HoLEP dissection procedure first described by York and colleagues in 2017 [16].

To the best of our knowledge, no studies have been published thus far utilizing top-down HoLEP to treat recurrent BPH following a prior TURP. This study aimed to evaluate the safety and urinary functional outcomes of top-down HoLEP in patients that previously underwent TURP for the management of BPH compared to patients with no history of surgical intervention of the prostate (nonrecurrent BPH) cases.

## 2. Materials and Methods

After obtaining the approval from the Research Ethics Board, we performed a retrospective review of a prospectively collected database of patients who underwent HoLEP at our institution between October 2017 and November 2021. From October 2017 to December 2020, we used a 100 W holmium:YAG laser (VersaPulse PowerSuite™, Lumenis, Yokneam, Israel). Afterward, a 120-W MOSES™ (Lumenis, Yokneam, Israel) was utilized from December 2020 to November 2021. A 550 *μ*m laser fibre and a 28-F continuous flow resectoscope (Karl Storz SE & Co., KG, Tuttlingen, Germany) were used for both techniques. The primary laser settings for enucleation were 2 J and 40 Hz, with 2 J and 20 Hz on the secondary laser foot-pedal for hemostasis. All procedures were performed by a single urologist (H.E), a HoLEP expert.

Preoperative evaluation included general patient demographics, a complete medical history, physical examination (including a digital rectal exam), a history of urinary retention, and prior prostate procedures. Symptom assessment with the International Prostate Symptom Score (IPSS) and quality of life (QoL) questionnaires was completed. All patients underwent basic laboratory workup, prostate-specific antigen (PSA) testing, uroflowmetry, a PVR bladder scan, and a transrectal ultrasound for prostate volume estimation.

A preoperative biopsy was performed on patients with PSA values above normal and/or abnormal digital rectal exam (DRE) findings to exclude prostate cancer. In group I, cystoscopy was performed to confirm that the cause of recurrent symptoms was due to regrowth or residual adenoma rather than a urethral stricture or bladder neck contracture.

We recorded the surgical parameters, including enucleation time, morcellation time, laser energy, resected weight, and intraoperative complications. Postoperative complications included clot retention, the need for a blood transfusion, urethral strictures, and bladder neck contraction. A detailed history of involuntary urine leakage while sneezing or coughing and the use of pads to prevent wetting were used to evaluate stress urinary incontinence (SUI). Additionally, SUI was clinically assessed by instructing the patient to cough with a full bladder and observing the passage of urine. All patients had postoperative follow-ups at 1, 3, 6, and 12 months. Our evaluation included the IPSS, QoL assessment, *Q*_max_, and PVR. PSA blood testing was conducted at 3 months.

### 2.1. Surgical Technique

All HoLEP procedures were performed by a single surgeon (H.E) using the top-down technique described in a previous publication [17]. Briefly, one posterior groove is created at either the 5 or 7-o'clock position up to the verumontanum. Afterward, the anterior commissure mucosa is incised at 2 J/20 Hz starting from the bladder neck at the 12-o'clock position. The incision is deepened to separate the area between the right and left adenoma, until reaching the surgical capsule. Once the plane between the adenoma and the surgical capsule is created, a top-down lateral lobe dissection is performed and extended anteroposteriorly towards the apical adenoma at 6 o'clock. Once the surgeon reaches the bladder neck at the 6 o'clock position, the remaining attachment between the adenoma and surgical capsule is cautiously separated to avoid injuring the ureteric orifices at the bladder neck from lateral to medial.

### 2.2. Statistical Analyses

The Statistical Package for the Social Sciences (SPSS® IBM®) version 26 was used in data collection and statistical analysis. Categorical parameters were presented in numbers and percentages and evaluated using the chi-squared test. Whereas, continuous data were mentioned in median and range and analyzed by the Mann–Whitney *U* test. A *p* value < 0.05 was considered statistically significant.

## 3. Results

A total of 269 patients were included in the study. Group I consisted of 68 patients with recurrent BPH post-TURP, while group II included 201 patients. Eighteen patients in group I (26.4%) underwent more than one TURP prior to HoLEP, and seven patients (10.3%) had more than two TURPs. None of the patients in our cohort had prior interventions apart from TURP.

The median patient age was 73.7 versus 71.6 years, and the median prostate volume was 122 versus 106 cc in groups I and II, respectively (Table 1). Other baseline demographics in terms of the indication for HoLEP, median preoperative PSA (ng/mL), median preoperative IPSS, median preoperative QoL, median preoperative maximum flow rate (*Q*_max_ (mL/sec)), and median preoperative PVR (cc) were comparable between both groups (*p* values >0.05) (Table 1).

There were no significant differences in the perioperative parameters between the two groups (Table 2). The median enucleation time for the recurrent BPH group (67.5 minutes (25–200)) was not significantly longer than the nonrecurrent group (60 minutes (19–165)) (*p*=0.25). Other operative outcomes, including morcellation time, enucleation efficiency, laser energy used, and resected weight, showed no significant difference between both groups. There were no significant differences in hospitalization and catheterization times between the two groups (*p* > 0.05).

No intraoperative complications were recorded for either group, apart from two patients (one from each cohort) with a simple bladder mucosal injury. There were no major complications among the cohorts according to the modified Clavien–Dindo classification system. One patient from group I that was on coumadin required a postoperative blood transfusion (Clavien II) (Table 2).

One patient (1.5%) in group I and five patients (2.5%) in group II developed clot retention a few days after the surgery (*p*=0.24). The patients were readmitted for clot evacuation using a 3-way catheter (Clavien I) (Table 2).

Two patients (one from each cohort) were hospitalized for 48 hours due to postoperative fever (Clavien II). Two patients (2.9%) in group I and eight (4%) in group II had a failed TOV postoperatively. All patients were successfully void within 7 days after discharge. Six patients in group I (8.8%) and twenty-one patients in group II (10.4%) were diagnosed with prostate cancer in the HoLEP specimen. Most of these patients had adenocarcinoma 3 + 3.

Late postoperative complications are shown in Table 2, with no significant difference between the two groups (*p* > 0.05). Urethral stricture or meatal stenosis occurred in 3 (4.4%) and 4 (2%) patients in groups I and II, respectively (Table 2). There was no reoperation due to persistent symptoms related to residual adenoma; however, one patient in group II developed a bladder neck contracture 18 months postoperatively, which was managed with a laser bladder neck incision.

The subjective and objective parameters significantly improved immediately after surgery compared to baseline parameters. During the follow-up period of up to 12 months, there was no difference between the two groups in terms of IPSS, QoL, *Q*_max_, and PVR (Table 3). One patient from group II had a PVR of 790 mL at one-year follow-up. The patient's PVR ranged from 200 to 300 mL at 1, 3, and 6 months' follow-up. The urodynamic study performed at one-year follow-up showed a hypotonic bladder, and the patient was started on clean intermittent catheterization (CIC).

Compared with the preoperative values, there were significant reductions in PSA levels after the procedure, with no significant difference between the groups. At 3 months' follow-up, two patients in group I (2.9%) and three patients in group II (1.5%) experienced SUI. At the last follow-up visit, one patient from group I presented with persistent SUI. Other SUI patients showed complete resolution at one-year follow-up. During the procedure, the surgeon did not have any difficulties identifying the surgical capsule, and the top-down technique was performed without shifting to traditional HoLEP technique.

## 4. Discussion

TURP is the standard surgical treatment modality for BPH management. However, the long-term durability of TURP may be a postoperative concern due to insufficient resection of the BPH adenoma. When evaluating minimally invasive technology for BPH, it is imperative to consider the retreatment rate, particularly with increasing patient age and comorbidities during the follow-up [11].

To the best of our knowledge, this is the first study to evaluate the role of top-down HoLEP in recurrent BPH following a previous TURP. Our study compared 68 patients with a history of previous prostate surgery with 201 patients with no prior surgery using the top-down HoLEP technique.

Enucleation time depends on various factors, including prostate size, prostate configurations, the presence of multiple nodules, degree of vascularity, and the surgeon's experience [9]. In our study, the median prostate volume was 122 cc in the recurrent BPH group versus 106 cc in the nonrecurrent group. The median enucleation time for the recurrent BPH group was 67.5 minutes, which was not significantly longer than the nonrecurrent group at 60 minutes. Krambeck and colleagues evaluated the outcomes of 37 patients that underwent HoLEP after previous transurethral prostate surgery. The mean prostate volume was 93.6 mL, which is comparable to our cohort [10]. Similarly, they found no significant differences in the perioperative parameters, including enucleation time (46 vs. 45 minutes) or resected tissue weight (61.7 vs. 63.9 g) between both groups.

In another study by Enikeev's group [12], operative time was slightly longer in the recurrent group compared to the primary HoLEP group by 3 minutes (50 vs. 47 min), although there was no significant difference. Similar to other studies, we did not face any challenges in identifying the capsule or dissection planes [10, 18, 19].

Generally, enucleation efficiency is considered a reliable method to measure operative difficulties. In the present study, the median enucleation efficiency of the recurrent BPH group was 1.27 g/min compared to 1.3 g/min for the nonrecurrent group. These results are similar to those previously reported by Krambeck's group (1.32 vs. 1.36 g/min) [10]. This indicates that the enucleation efficiency in the recurrent BPH group was not inferior to that of the nonrecurrent HoLEP cohort. Some technical modifications have been introduced to the conventional HoLEP aiming to reduce enucleation time, shorten the learning curve, and improve continence status. Despite similar functional outcomes, the impact of these modifications on enucleation time is controversial.

Rucker and colleagues compared the outcomes of three enucleation techniques and found that en bloc and two-lobe enucleation were significantly faster than the three-lobe technique with respect to enucleation time [20]. In contrast, Enikeev et al.'s comparative analysis of the en bloc and two-lobe techniques demonstrated no difference in the duration of the surgery [21].

In our study, postoperative functional outcomes significantly improved immediately after surgery compared to baseline parameters. During the follow-up period of up to 12 months, there was no significant difference between the two groups in terms of IPSS, QoL, *Q*_max_, and PVR. HoLEP postoperative functional parameters suggested significant and durable improvement similar to previous studies regardless of prior surgery [9, 10, 12, 19]. The development of transient SUI post-HoLEP is multifactorial [22].

In our study, the incidence of transient stress incontinence at 3 months was 2.9% in the recurrent group versus 1.5% in the nonrecurrent group. One patient from the recurrent group had persistent urinary incontinence at a one-year follow-up.

Similarly, in Krambeck group's study, postoperative SUI was reported as 3% in the secondary group and 4% in the primary group [10]. Two other studies reported similar rates of postoperative SUI ranging from 4.5 to 6.5% [9, 12]. The postoperative complications were comparable with no significant difference for both groups using the Clavien–Dindo grading system. These findings are similar to other retrospective studies in the literature [10, 12].

HoLEP for recurring adenoma has a low retreatment rate (0.8%) [9]. The retreatment rate of secondary HoLEP to manage recurring adenoma occurs at a rate of 1.3%, particularly in the early learning curve [9]. None of the patients in our cohort required repeat interventions for recurrent LUTS secondary to prostate regrowth until their last follow-up.

Results of the first RCT that compared HoLEP to TURP found that none of the patients that initially underwent HoLEP needed surgery for recurrent BPH adenoma at seven years follow-up [2]. Similarly, Krambeck's group found that none of the patients in the primary or secondary cohorts required a secondary procedure for recurrent adenoma [10].

Our study has some limitations, including its retrospective nature and that it is a single-center experience with unequal sample sizes in the two groups. Moreover, our study did not evaluate the impact of repeated transurethral surgery on sexual function. Additional studies with larger sample sizes and more extended follow-up periods are warranted to determine the feasibility of HoLEP in treating recurrent BPH after various minimally invasive procedures.

## 5. Conclusion

Top-down HoLEP is safe and effective with comparable urinary functional outcomes for managing recurrent and nonrecurrent cases of BPH. Patients with a history of prostate surgery can be counselled that their prior transurethral procedure does not reduce the benefits of HoLEP.

## Figures and Tables

**Table 1:** Preoperative parameters.

Parameter	Post-TURP Top-down HoLEP *n*=68	Nonrecurrent Top-down HoLEP *n*=201	*P* value
Age at surgery, median (range), years	73.7 (58.4–90.2)	71.6 (51.8–93.3)	0.10
Prostate size, median (range), cc	122 (50–273)	106 (42–300)	0.45
Urine retention, *n* (%)	34 (50)	81 (40.3)	0.16
IPSS, median (range)	22 (12–31)	23 (5–35)	0.23
QoL, median (range)	5 (2–6)	5 (1–6)	0.81
*Q *_max_, median (range), mL/s	12 (4.5–17.6)	7.7 (1.4–67)	0.10
PVR, median (range), mL	220 (0–1500)	328 (2.2–2600)	0.10
PSA, median (range), ng/dL	5.7 (1.3–79)	5 (0.4–27.5)	0.33

*n*, number; IPSS, International Prostate Symptom Score; QoL, quality of life; *Q*_max_, maximum flow rate; PVR, postvoid residual; PSA, prostate-specific antigen.

**Table 2:** Perioperative parameters.

Parameter	Post-TURP Top-down HoLEP *n* = 68	Nonrecurrent Top-down HoLEP *n* = 201	*P* value
Enucleation time, median (range), minutes	67.5 (25–200)	60 (19–165)	0.25
Morcellation time, median (range), minutes	12 (1–58)	11 (2–55)	0.16
Resected tissue, median (range), grams	87 (18–242)	75 (20–240)	0.15
Enucleation efficiency, median (range) grams/minute	1.27 (0.5–2.55)	1.3 (0.28–3.19)	0.97
Energy, median (range), KJ	119.9 (37.5–325.1)	110.2 (45.1–355.4)	0.08
Intraoperative complications, *n* (%)	1 (1.5)	1 (0.5)	0.42
Fever, urinary tract infection *n* (%)	1 (1.5)	1 (0.5)	0.42
Postop clot retention, *n* (%)	1 (1.5)	5 (2.5)	0.24
Postop blood transfusion, *n* (%)	1 (1.5)	0 (0)	0.08
Postop stricture/meatal stenosis *n* (%)	3 (4.4)	4 (2)	0.07
Hospital stay, median (range), hours	24 (3–48)	24 (3–72)	0.15
Catheter duration, median (range), hours	24 (3–48)	24 (2–336)	0.62

*n*, number; KJ, kilojoule.

**Table 3:** Outcomes at 1, 3, 6, and 12 months follow-up.

Parameter	Post-TURP Top-down HoLEP *n* = 68	Nonrecurrent Top-down HoLEP *n* = 201	*P* value
Outcomes at 1 month			
Number of patients, *n* (%)	65/68 (95.6)	192/201 (95.5)	—
IPSS, median (range)	6 (0–25)	7 (0–25)	0.24
QoL, median (range)	1 (0–6)	2 (0–6)	0.11
*Q*_max_, median (range), mL/s	24.1 (5.3–65)	24 (5.8–73.3)	0.90
PVR, median (range), mL	41 (0–400)	46.5 (0–393)	0.70
Stress incontinence, *n* (%)	5 (7.3)	11 (5.5)	0.59

Outcomes at 3 months			
Number of patients, *n* (%)	63/68 (92.6)	183/201 (91)	—
IPSS, median (range)	5 (0–23)	4 (0.27)	0.73
QoL, median (range)	1 (0–6)	1 (0–6)	0.08
*Q*_max_, median (range), mL/s	21.5 (3–47.3)	21.7 (7.8–54.2)	0.97
PVR, median (range), mL	56 (0–225)	44 (0–400)	0.88
Stress incontinence, *n* (%)	2 (2.9)	3 (1.5)	0.44
% PSA reduction, median (range)	88.5 (−67.1–97.9)	87.4 (−27.3–98.9)	0.63

Outcomes at 6 months			
Number of patients, *n* (%)	61/68 (89.7)	171/201 (85.1)	—
IPSS, median (range)	3.5 (0–17)	4 (0–23)	0.27
QoL, median (range)	0.5 (0–4)	1 (0–6)	0.68
*Q*_max_, median (range), mL/s	24.8 (13.4–47.8)	23.7 (6.9–69)	0.50
PVR, median (range), mL	38 (0–285)	47 (0–480)	0.80
Stress incontinence, *n* (%)	1 (1.5)	2 (0.9)	0.75

Outcomes at 12 months			
Number of patients, *n* (%)	59/68 (86.8)	168/201 (83.6)	—
IPSS, median (range)	2.5 (0–17)	3 (0–17)	0.65
QoL, median (range)	0.5 (0–3)	0 (0–4)	0.54
*Q*_max_, median (range), mL/s	28.3 (9.6–51.4)	24.3 (5.2–65.4)	0.82
PVR, median (range), mL	28.5 (0–185)	36 (0–790)	0.63
Stress incontinence, *n* (%)	1 (1.5)	0 (0)	0.09

*n*, number; IPSS, International Prostate Symptom Score; QoL, quality of life; *Q*_max_, maximum flow rate; PVR, postvoid residual; PSA, prostate-specific antigen.

## Data Availability

The data used to support the findings of this study are available from the corresponding author upon request.
